# Emergency administration of fibrinogen concentrate for haemorrhage: systematic review and meta-analysis

**DOI:** 10.1186/s13017-023-00497-5

**Published:** 2023-03-30

**Authors:** Yuki Itagaki, Mineji Hayakawa, Yuki Takahashi, Satoshi Hirano, Kazuma Yamakawa

**Affiliations:** 1 Department of Surgery, Kushiro City General Hospital, 1-12 Shunkodai, Kushiro, Hokkaido 085-0822 Japan; 2 Department of Emergency Medicine, Hokkaido University Hospital, Sapporo, Hokkaido Japan; 3 Department of Gastroenterological Surgery II, Faculty of Medicine, Hokkaido University, Sapporo, Hokkaido Japan; 4 Department of Emergency and Critical Care Medicine, Osaka Medical and Pharmaceutical University, Osaka, Japan

**Keywords:** Coagulopathy, Fibrinogen concentrate, Haemorrhage, Transfusion

## Abstract

**Introduction:**

The occurrence of massive haemorrhages in various emergency situations increases the need for blood transfusions and increases the risk of mortality. Fibrinogen concentrate (FC) use may increase plasma fibrinogen levels more rapidly than fresh-frozen product or cryoprecipitate use. Previous several systematic reviews and meta-analyses have not effectively demonstrated FC efficacy in significantly improving the risk of mortality and reducing transfusion requirements. In this study, we investigated the use of FC for haemorrhages in emergency situations.

**Methods and analysis:**

In this systematic review and meta-analysis, we included controlled trials, but excluded randomized controlled trials (RCTs) in elective surgeries. The study population consisted of patients with haemorrhages in emergency situations, and the intervention was emergency supplementation of FC. The control group was administered with ordinal transfusion or placebo. The primary and secondary outcomes were in-hospital mortality and the amount of transfusion and thrombotic events, respectively. The electronic databases searched included MEDLINE (PubMed), Web of Science, and the Cochrane Central Register of Controlled Trials.

**Results:**

Nine RCTs in the qualitative synthesis with a total of 701 patients were included. Results showed a slight increase in in-hospital mortality with FC treatment (RR 1.24, 95% CI 0.64–2.39, *p* = 0.52) with very low certainty of the evidence. There was no reduction in the use of red blood cells (RBC) transfusion in the first 24 h after admission with FC treatment (mean difference [MD] 0.0 Unit in the FC group, 95% CI − 0.99–0.98, *p* = 0.99) with very low certainty of the evidence. However, the use of fresh-frozen plasma (FFP) transfusion significantly increased in the first 24 h after admission with FC treatment (MD 2.61 Unit higher in the FC group, 95% CI 0.07–5.16, *p* = 0.04). The occurrence of thrombotic events did not significantly differ with FC treatment.

**Conclusions:**

The present study indicates that the use of FC may result in a slight increase in in-hospital mortality. While FC did not appear to reduce the use of RBC transfusion, it likely increased the use of FFP transfusion and may result in a large increase in platelet concentrate transfusion. However, the results should be interpreted cautiously due to the unbalanced severity in the patient population, high heterogeneity, and risk of bias.

**Supplementary Information:**

The online version contains supplementary material available at 10.1186/s13017-023-00497-5.

## Background

The occurrence of massive haemorrhages in various emergency situations, such as severe trauma, major surgeries, and postpartum, increases the need for blood transfusions and the risk of mortality [1–3]. Fibrinogen levels tend to deteriorate faster than other coagulation factors in conditions such as severe trauma, surgical bleeding, and postpartum [4–7], highlighting the importance of aggressively supplementing fibrinogen haemostasis [8, 9]. Fibrinogen can be supplemented with fresh-frozen plasma (FFP), cryoprecipitate, or fibrinogen concentrate (FC). FC has several advantages over these alternatives, including the fact that it does not require a thawing process or ABO compatibility confirmation [10]. Furthermore, FC may increase plasma fibrinogen levels more rapidly than FFP or cryoprecipitate [11] and reduce transfusion volume and the risk of immunogenic or infectious complications [12]. These features make FC a promising option for the management of haemorrhage.

Several systematic reviews and meta-analyses of randomized controlled trials (RCTs) examining the use of major surgery have been published [13–16]. However, these reviews primarily focused on elective surgeries and did not consider emergency settings. The use of FC has been shown to rapidly supplement fibrinogen levels [17], making it a potential option in emergency situations. In 2017, Innerhofer et al. [18] conducted an RCT that demonstrated the superiority of FC supplementation with other coagulation factor concentrates, compared to FFP in reducing the need for massive transfusion. However, a meta-analysis by Stabler et al. in 2020 found that FC did not significantly improve mortality rates or reduce transfusion requirements in trauma-related RCTs [19]. This meta-analysis should be interpreted with caution due to the small sample sizes and low quality of the included evidence.

There is a lack of comprehensive research on the efficacy of FC administration in emergency situations, including trauma and massive haemorrhage. In order to address this gap in knowledge, we conducted a systematic review and meta-analysis of studies examining the safety and effectiveness of FC administration in emergency settings, compared to traditional transfusion strategies. Our aim was to assess the early supplementation of fibrinogen in emergent massive haemorrhage using FC.

## Methods

### Protocol registration

This study protocol has been published [20] and registered in the UMIN (UMIN registration number: UMIN0000415989, URL https://upload.umin.ac.jp/cgi-open-bin/ctr_e/ctr_view.cgi?recptno=R000047487). This systematic review and meta-analysis was reported according to The Preferred Reporting Items for Systematic Reviews and Meta-Analyses Protocols guidelines [21].

### Database search

To retrieve relevant articles, we performed a literature search in the following major electronic bibliographic databases: MEDLINE (PubMed), Web of Science, and the Cochrane Central Register of Controlled Trials. The details of the search strategy are available in the protocol of this systematic review and meta-analysis [20].

### Types of studies

In this systematic review and meta-analysis, we included controlled trials (including ongoing RCTs and other controlled trials) published until October 17, 2020. Studies were excluded if they did not clearly report the population, treatment, or outcomes of interest. We also excluded RCTs on elective surgeries and animal studies. No language restrictions were be applied. For non-English language publications, we utilized appropriate translation services. Our focus was on evaluating the effect of FC on uncontrolled bleeding in emergency situations.

### Study population

This study included patients admitted to the hospital with emergency haemorrhage. The aetiologies of the haemorrhages included trauma, postpartum bleeding, cardiovascular diseases, and emergency surgery. Elective surgery patients were excluded from analysis. No geographical restrictions were placed on the study sample.

### Intervention and control

The focus of this study was the use of emergency fibrinogen supplementation as an intervention for haemostasis. The control groups were administered standard transfusion treatments (i.e. FFP) or placebo. No restrictions were placed on the type, amount, or timing of FC administration in the review.

### Outcomes

The primary outcome measure was all-cause in-hospital mortality. Secondary outcome measures included the volume of transfused blood within the first 24 h, blood loss within the first 24 h, thrombotic events (i.e. deep venous thromboses, pulmonary embolization, myocardial infarctions, strokes), multiple organ failure, length of intensive care unit (ICU) stay, and length of hospital stay.

### Selection of studies

Citations were stored and duplicates were removed using EndNote X9.3.3 (Thomson Reuters, Toronto, Ontario, Canada). The systematic review process was conducted using Rayyan software [22]. Two reviewers (YI and YT) independently screened the titles and abstracts of retrieved studies using the search strategy to identify those that potentially met the inclusion criteria. The full texts of these potentially eligible studies were retrieved and independently assessed by the two reviewers (YI and YT). Any disagreement regarding study eligibility was resolved through consultation with a third reviewer (KY).

### Data extraction

A standardized, pre-piloted form was used to extract data from the included studies in order to assess the quality of the studies and the methods of data synthesis. The extracted information included the following variables: study setting, study population, baseline characteristics of the participants, details of the interventions and control conditions, study methodology, outcomes, and assessments of risks bias. Two independent reviewers (YI and YT) extracted the data independently, and any discrepancies were resolved through discussion with the third author (KY).

### Assessment of risk of bias in individual studies

The two independent reviewers (YI and YT) assessed the risk of bias and methodological quality of the articles, and any disagreements were resolved by discussion with a third reviewer (KY). The risk of bias in individual studies was evaluated using uniform criteria based on the tool for assessing the risk of bias in randomized trials (RoB2) [23]. This included assessment of (1) random sequence generation, (2) allocation concealment, (3) blinding of participants and personnel, (4) blinding of outcome assessment, (5) incomplete outcome data, (6) selective reporting, and (7) other bias for each study.

### Data summary

A meta-analysis was performed when data were available in one or more trials, following the guidelines of the “Cochrane Handbook for Systematic Reviews of Interventions [24].” For binary variables, risk ratios or odds ratios were expressed as point estimates with 95% confidence intervals (CIs). Continuous variables, such as length of ICU stay, were expressed as mean differences with 95% CIs and *p* values. Mean and standard deviation were estimated from sample size, median, and interquartile range using appropriate methods [25, 26].

### Data synthesis

Findings from the included studies were estimated using a random-effects model. This model considers statistical heterogeneity and provides a more conservative estimate of the pooled effect size than a fixed-effects model. No multiple imputations were performed for missing data, data synthesis, or analysis of randomized trials.

All statistical analyses, including assessment of risk of bias within and across studies were conducted using Review Manager, Version 5.4. (RevMan; The Cochrane Collaboration 2019, The Nordic Cochrane Centre, Copenhagen, Denmark) [27]. A *p* value of < 0.05 was considered statistically significant.

### Assessment of heterogeneity

Statistical heterogeneity was assessed using the Mantel–Haenszel *χ*^2^ test and the *I*^2^ statistic (with *I*^2^ values > 50% indicating significant heterogeneity) [28]. The presence of clinical heterogeneity was considered in the decision to conduct a quantitative synthesis of data or perform sensitivity analyses.

### Sensitivity analysis

We examined the robustness of this meta-analysis by conducting sensitivity analysis according to the different components of the Cochrane risk of bias tool. We also conducted an analysis in which studies with a high overall risk of bias judgement were excluded.

### Subgroup analysis

Subgroup analyses were performed according to the type of haemorrhage experienced by the patient.

### Rating the strength of evidence using the GRADE approach

The two authors (YI and YT) assessed the strength of evidence independently, using the Grading of Recommendations Assessment, Development and Evaluation (GRADE) approach [29]. The quality of evidence was assessed for each outcome and categorized as high, moderate, low, or very low according to the GRADE approach.

### ***Patient and public involvement***

Patients and the general public were not involved in the design of this systematic review and meta-analysis.

## Results

### Search results and characteristics of included trials

**Fig. 1:**
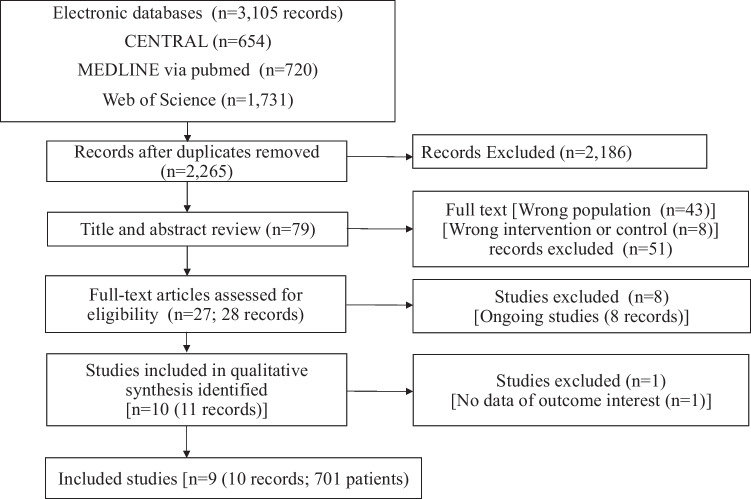
PRISMA flowchart

**Fig. 2:**
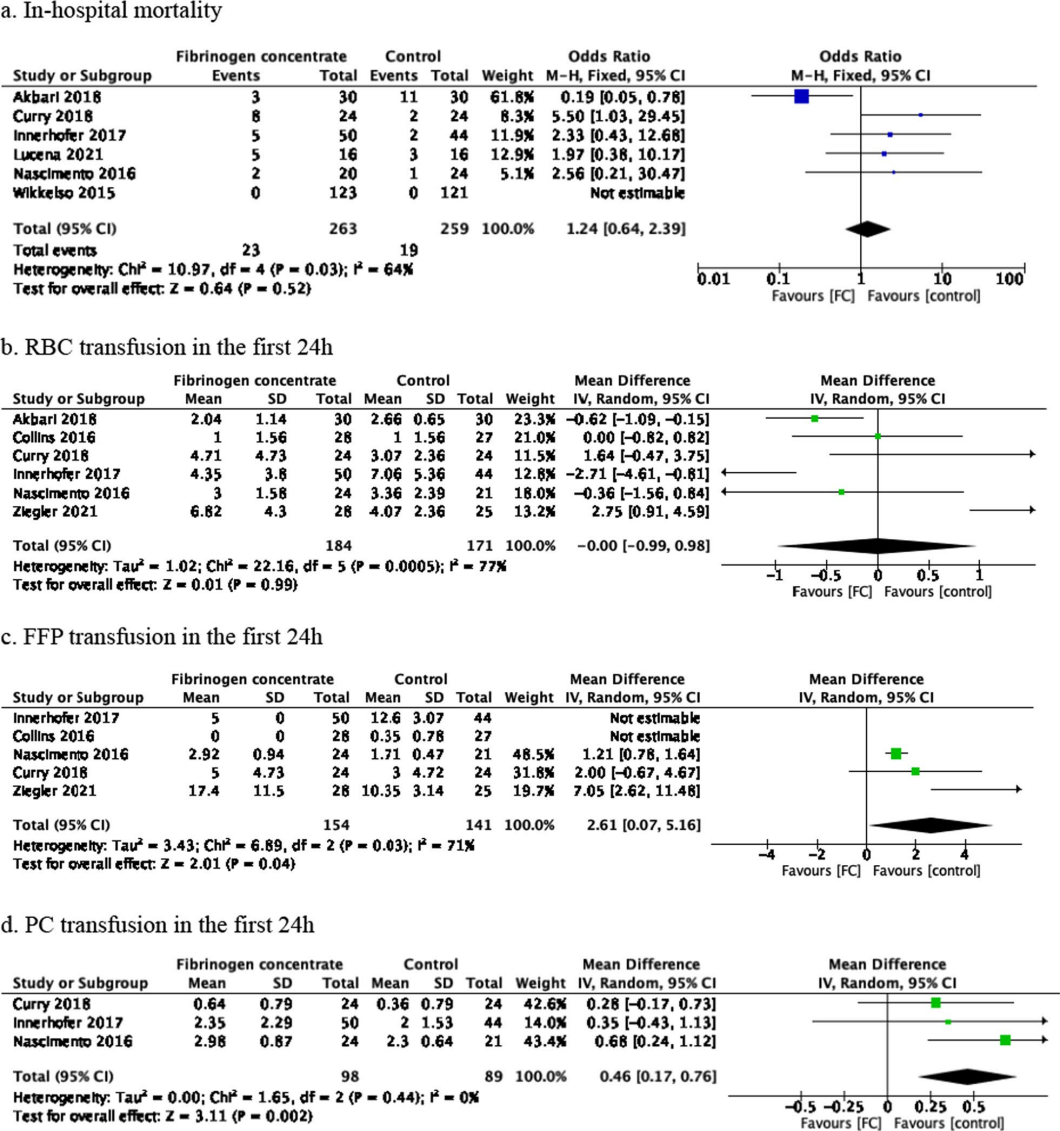
Forest plots for primary and secondary outcomes

After removing duplicates, 2265 records were identified during the search conducted in November 2020. Twenty-eight full-text articles were identified and assessed for their eligibility, and nine RCTs with a total of 701 patients were included in the qualitative synthesis (see Fig. 1. For PRISMA flowchart). Table 1 summarizes the published studies included in the synthesis. Data from seven trials were analysed and are shown in Fig. 2 and Additional file 1: Figs. S1–S4. The risk of bias assessment is summarized in Additional file 1: Figs. S5. The summary of findings is shown in Table 2.

**Table 1:** The published studies included in the data synthesis

Study	Region	Study participants	Number of participants (male, female)	Age, (%) or Mean (SD) or median [25–75%IQR]	Intervention	Reported outcome of interest	Primary outcome of the original study	ISS
Sabouri [35]	Iran	Isolated traumatic brain injury	71 [54, 17]	No information	Calculated amounts of FC	Length of ICU stay, Length of hospital days	Mortality rate	Isolated head injury with on-admission GCS < 9
Lucena [34]	Brazil	Trauma	32 [4, 28]	Placebo 40.2 (15.7) FC 44 (16.3)	50 mg/kg of body weight	In-hospital mortality, Thrombosis events, Length of ICU stay, Length of hospital days	Feasibility of FC administration	ISS≧ 15
Ziegler [32]	International	Trauma	53 [44, 9]	Placebo 54 [37–56] FC 46 [34.5–58]	3 g of the study drug was given in patients with bodyweight 30 to 60 kg, 4.5 g in patients with bodyweight 60 to 90 kg and 6 g in patients with bodyweight 90 to 120 kg	The change in FIBTEM MCF on arrival at the ED, the need for volume and requirements for transfusion, thromboembolic complications	The change in FIBTEM MCF on arrival at the ED	Placebo 16 [16–34], FC 25 [16–36]
Akbari [30]	Iran	Trauma	60 [49, 11]	Placebo 18–39.9(76.7%), 40–59.9(13.3%), 60 ≤ (10.0%) FC 18–39.9(73.4%), 40–59.9(10%), 60 ≤ (16.6%)	2 g of FC	In-hospital mortality, RBC transfusion 24 h, Thrombotic events, Multiple organ failure, Length of hospital day	Mortality	Placebo 19 ± 4.3 FC 19.3 ± 4.3 (mean ± SD)
Curry [11]	UK	Trauma	48 [39, 9]	Placebo 38.7 (12.6) FC 38.1 (26.8)	6 g of FC	In-hospital mortality, RBC transfusion 24 h, FFP transfusion 24 h, PC transfusion 24 h	Feasibility	Placebo 29 [11, 22–33], FC 34 [24–43]
Innerhofer [18]	Austria	Trauma	94 [70, 24]	Placebo 40.0 (17.7) FC 40.7 (24.5)	50 mg/kg of FC	In-hospital mortality, RBC transfusion 24 h, FFP transfusion 24 h, PC transfusion 24 h, Thrombotic events, Multiple organ failure, Length of ICU stay, Length of hospital day	Multiple organ failure	FFP 30 [24–45], FC 35 [29–42]
Nascimento [33]	Canada	Trauma	44 [8, 11]	Placebo 48 [19–78] FC 28 [19–88]	6 g of FC	In-hospital mortality, RBC transfusion 24 h, FFP transfusion 24 h, PC transfusion 24 h, Thrombotic events	Feasibility	Placebo 23 [18–29], FC 25 [19–29]
Collins [17]	UK	Postpartum haemorrahge	55 [0, 55]	Placebo 30.8 [19–42] FC 33.5 [20–48]	FC dose was calculated according to Fibtem A5, ideal body wight for height	RBC transfusion 24 h, FFP transfusion 24 h, Bood loss within 24 h, Thrombotic events, Length of hospital day	The number of allogeneic blood products (RBC, FFP, cryoprecipitate, platelets) infused after study medication until hospital dishcarge	NA
Wikkelso [31]	Denmark	Postpartum haemorrahge	244 [0, 244]	No information	2 g of FC	In-hospital mortality, Thrombotic events	RBC transfusion during a 6 week follow-up period postpartum	NA

Fibrinogen concentrate; FC, Injury Severity Score; ISS, maximum clot firmness in the FIBTEM assay; FIBTEM MCF

**Table 2:** Summary of findings

Fibrinogen concentrate compared to placebo for emergency haemorrhagic event						
Patient or population: emergency haemorrhagic event						
Setting: RCT						
Intervention: Fibrinogen concentrate						
Comparison: placebo						
Outcomes	Anticipated absolute effects* (95% CI)		Relative effect (95% CI)	No of participants (studies)	Certainty of the evidence (GRADE)	Comments
	Risk with placebo	Risk with Fibrinogen concentrate				
In-hospital mortality	66 per 1000	82 per 1000 (42 to 157)	RR 1.24 (0.64 to 2.39)	522 (6 RCTs)	Very low^a,b^	The evidence suggests fibrinogen concentrate results in a slight increase in in-hospital mortality
RBC transfusion 24 h	The mean RBC transfusion 24 h ranged from 0.62 to 6.82 Unit	MD 0 Unit (0.99 lower to 0.98 higher)	–	355 (6 RCTs)	Very low^a,b,c^	Fibrinogen concentrate may reduce/have little to no effect on RBC transfusion 24 h but the evidence is very uncertain
FFP transfusion 24 h	The mean FFP transfusion 24 h ranged from 0 to 17.4 Unit	MD 2.61 Unit higher (0.07 higher to 5.16 higher)	–	295 (5 RCTs)	Low^b,c^	Fibrinogen concentrate probably increases FFP transfusion 24 h
PC transfusion 24 h	The mean PC transfusion 24 h ranged from 0.64 to 2.98 Unit	MD 0.46 Unit higher (0.17 higher to 0.76 higher)	–	187 (3 RCTs)	Moderate^b^	Fibrinogen concentrate likely results in a large increase in PC transfusion 24 h
Blood loss within 24 h	The mean blood loss within 24 h was **221.8** mL	MD 171 mL lower (400.35 lower to 58.35 higher)	–	55 (1 RCT)	Low^d^	Fibrinogen concentrate may result in little to no difference in blood loss within 24 h
Thrombotic events	53 per 1000	38 per 1000 (17 to 83)	RR 0.71 (0.32 to 1.58)	529 (6 RCTs)	Very low^a,b^	Fibrinogen concentrate likely results in little to no difference in thrombotic events
Multiple organ failure	500 per 1000	270 per 1000 (90 to 780)	RR 0.54 (0.18 to 1.56)	154 (2 RCTs)	Low^a,b^	Fibrinogen concentrate may result in a slight reduction in multiple organ failure

*The risk in the intervention group (and its 95% confidence interval) is based on the assumed risk in the comparison group and the relative effect of the intervention (and its 95% CI)

*CI*—Confidence interval; *MD*—Mean difference; *RR*—Risk ratio

GRADE Working Group grades of evidence

*High certainty* We are very confident that the true effect lies close to that of the estimate of the effect

*Moderate certainty* We are moderately confident in the effect estimate: the true effect is likely to be close to the estimate of the effect, but there is a possibility that it is substantially different

*Low certainty* Our confidence in the effect estimate is limited: the true effect may be substantially different from the estimate of the effect

*Very low certainty* We have very little confidence in the effect estimate: the true effect is likely to be substantially different from the estimate of effect

^a^Due to moderate weight of high risk of bias

^b^Due to low events number for optimal information size

^c^Due to high heterogeneity

^d^Due to very low events number for optimal information size

### Primary outcomes

#### In-hospital mortality

Data from five trials indicated that FC as a treatment for emergency haemorrhagic events may result in a slight increase in in-hospital mortality (RR 1.21, 95% CI 0.49–3.00, *p* = 0.68). However, due to the moderate weight of high risk of bias and low number of events, the certainty of this evidence is very low. Statistical heterogeneity was also observed (Tau^2^ = 0.68; *I*^2^ = 54%). In order to address this heterogeneity in in-hospital mortality, a sensitivity analysis was conducted (Additional file 1: Fig. S2a). When considering the high risk of bias, the risk ratio for in-hospital mortality was 1.21 (95% CI 0.49–3.00, *p* = 0.68). Further details on risk of bias can be found in Additional file 1: Fig. S5.

### Secondary outcomes

#### RBC Transfusion in the first 24 h

The results from six clinical trials suggest that FC does not significantly reduce the need for RBC transfusion within the first 24 h after admission (MD 0.00 units lower in the FC group, 95% CI − 0.99–0.98, *p* = 0.99). However, the certainty of this evidence is very low due to moderate weight of high risk of bias, low number of events, and high levels of heterogeneity (Tau^2^ = 1.02; *I*^2^ = 77%). A sensitivity analysis was conducted with the removal of the high risk of bias study, resulting in a MD of 0.03 units for RBC transfusion in the first 24 h (95% CI − 0.55–0.62, *p* = 0.001) (Additional file 1: Fig. S2b). Subgroup analyses were also performed for each type of haemorrhage. Further details on risk of bias can be found in Additional file 1: Fig. S5.

#### FFP Transfusion in the first 24 h

The results from five clinical trials suggest that FC may increase the need for FFP transfusion in the first 24 h after admission (MD 2.61 units higher in the FC group, 95% CI 0.07–5.16, *p* = 0.04). The certainty of this evidence is low due to the low number of events. Statistical heterogeneity was also observed (Tau^2^ = 3.43; *I*^2^ = 71%) (see Fig. 2c). A sensitivity analysis was conducted with the removal of the high risk of bias study, resulting in a mean difference of 1.21 units for FFP transfusion (95% CI 0.76–1.67, *p* < 0.01) (see Additional file 1: Fig. S2c). Subgroup analyses were also performed for each type of haemorrhage.

#### PC transfusion in the first 24 h

The results of the study suggest that FC may result in a large increase in PC transfusion within the first 24 h after admission (MD 0.46 units higher in the FC group, 95% CI 0.17–0.76, *p* = 0.002). The certainty of this evidence is moderate due to the moderate number of events. No statistical heterogeneity was observed (Tau^2^ = 0.00; *I*^2^ = 0%) (Fig. 2d). Further details on risk of bias can be found in Additional file 1: Fig. S5.

#### Blood loss within first 24 h after admission

The results of a clinical trial suggest that FC may result in little to no difference in blood loss within the first 24 h. The mean blood loss within the first 24 h was 392.88 mL (MD 171 mL lower in the FC group, 95% CI − 400.35–58.35, *p* = 0.14). The risk of bias was low due to the low number of events. Heterogeneity was not applicable (see Additional file 1: Fig. S1a). Further details on risk of bias can be found in Additional file 1: Fig. S5.

#### Thrombotic events

The results from five clinical trials suggest that FC may result in little to no difference in thrombotic events. The risk ratio was 0.71 (95% CI 0.32–1.58, *p* = 0.4) with very low level of certainty due to the moderate weight of high risk of bias and low number of events. No statistical heterogeneity was observed (Tau^2^ = 0.00; *I*^2^ = 0%) (see Additional file 1: Fig. S1b). Further details on Risk of bias can be found in Additional file 1: Fig. S5.

#### Multiple organ failure

The results from two clinical trials suggest that FC may result in a slight reduction in the risk of multiple organ failure. The risk ratio was 0.54 (95% CI 0.18–1.56, *p* = 0.25) with a low risk of bias due to the moderate weight of high risk of bias and low number of events. Statistical heterogeneity was observed (Tau^2^ = 0.40; *I*^2^ = 57%) (see Additional file 1: Fig. S1c). Further details on the risk of bias can be found in Additional file 1: Fig. S5. The outcome of MOF was reported by Innerhofer et al. [18] and Akbari et al. [30]. In the RCT by Innerhofer, the Sequential Organ Failure Assessment (SOFA) score was reported daily in the ICU, with a score of 3 or more for at least two organ systems defined as multiple organ failure. Although they reported the rate of MOF, the specific SOFA points for the organ systems were not reported in their manuscript or supplementary materials. In the quasi-RCT by Akbari et al., the specific criteria for MOF were not defined.

#### Length of ICU stay

The results from one clinical trial suggest that FC may result in little to no difference in the length of ICU stay. The mean length of ICU stay was lower in the FC group (MD, − 2.22 days lower, 95% CI − 4.96–0.52, *p* = 0.11) with a very low risk of bias due to the moderate weight of high risk of bias and low number of events. Statistical heterogeneity was not applicable (see Additional file 1: Fig. S1d). Further details on risk of bias can be found in Additional file 1: Fig. S5.

#### Length of hospital stay

The results of two clinical trials suggest that FC may result in a slight reduction in the length of hospital day. The mean length of hospital stay was lower in the FC group (MD, − 1.08 days lower, 95% CI − 3.35–1.19, *p* = 0.35) with a very low risk of bias due to the moderate weight of high risk of bias and low number of events. Statistical heterogeneity was not observed (Tau^2^ = 3.11; *I*^2^ = 50%) (see Additional file 1: Fig. S1e). Further details on risk of bias can be found in Additional file 1: Fig. S5.

#### Subgroup analysis: Trauma setting

The results of the study indicate that the risk ratio for in-hospital mortality was 1.08 (95% CI 0.64–1.84, *p* = 0.77). The MD in RBC transfusion was – 0.35 units (95% CI − 0.76–0.05, *p* = 0.02). The MD in FFP transfusion was 1.24 units (95% CI 0.79–1.69, *p* < 0.01). The mean difference of PC transfusion in the first 24 h was 0.46 units (95% CI 0.17–0.76, *p* = 0.002). The RR for the thrombotic events was 0.67 (95% CI 0.30–1.51, *p* = 0.33) (Additional file 1: Fig. S3a–e).

#### Subgroup analysis: obstetrics setting

We found only two RCTs [17, 31]. In-hospital mortality was not analysed as the included RCTs reported no death. The MD of RBC transfusion was—0.09 (95% CI − 0.87 to − 0.69, *p* = 0.82). The MD of the FFP transfusion was not estimable due to no demand of FFP in the included trial. The risk ratio of the thrombotic events was 0.96 (95% CI 0.06–16.21, *p* = 0.98) (see Additional file 1: Fig. S4a, b).

## Discussion

In this systematic review and meta-analysis, a systematic search for RCTs in the emergency bleeding setting resulted in studies conducted in trauma and postpartum settings. FC did not significantly decrease in-hospital mortality or the amount of transfusion. However, FC may result in little to no difference in thrombotic events and a slight reduction in the risk multiple organ failure. It is important to note that due to the unbalanced severity of the patient population, high levels of heterogeneity, and risk of bias, these results should be interpreted cautiously.

In the present study, FC had no significant effect on reducing in-hospital mortality or RBC transfusion within the first 24 h after admission, though the evidence for this finding is very low. In the subgroup analysis for trauma patients, FC administration was associated with a slight decrease in the amount of RBC transfusion, but no significant difference in in-hospital mortality was observed. It is important to note that due to the high levels of heterogeneity among the studies and the short duration of treatment for the patients, the results may not be generalizable.

The demand of FFP and PC were controversial despite of FC administration. This may be in part due to the heterogeneity of the included studies. In the trauma setting, patients in the FC group had higher ISS scores [11, 18, 32, 33] and more severe haematological conditions than patients in the control group [11], which may have affected the amounts of FFP and PC transfusion. Additionally, the majority of the studies included in the meta-analysis were open-label RCTs, which may have resulted in more aggressive transfusion of these blood products in the FC group compared to the Control group [18, 30, 34, 35]. The RETIC trial by Innerhofer et al., found a significant reduction in FFP transfusion in the FC group compared to the control group [18] with a median 5 units in the FC group (IQR 5 to 5), versus 14 units in the control group (IQR 10 to 14) (*p* = 0.023). However, due to the similarity of the first and third quartiles of FFP administration, this trial could not be included in the meta-analysis. The lack of availability of this data may have impaired the quality of the meta-analysis.

The results of studies conducted in the trauma settings suggest that FC may slightly reduce the risk of multiple organ failure and the length of ICU and hospital stays. Despite the higher amount of transfusion in the FC group, these results may be due to the stabilizing effect of FC on the vital signs and organ damage of severely injured patients. However, as the amounts of crystalloids and measure of haemostasis were not obtained from the included studies. Pre-intervention platelet transfusion was not performed and the timing of PC transfusion in the first 24 h was not reported in the included studies [11, 18, 33]. Thus, more information is needed to fully understand the mechanisms underlying these effects. Further investigation is warranted.

This study has several limitations. The types of bleeding included in the analysis were limited, and the obstetrics subgroup had a lower number of events, resulting in no significant differences. The number of eligible patients and the amount of high-quality randomized controlled data were insufficient [4, 11, 32]. Furthermore, the specific haemostasis procedures used in the trauma setting RCTs were not described, and the supplementation of tranexamic acid or other coagulation factors was not precisely investigated in this meta-analysis. The studies included in the meta-analyses varied in terms of risk of bias, and particularly in the emergency setting, it is difficult to include a sufficient number of patients and control for patient characteristics in RCTs. While some of the studies were conducted in a double-blinded setting, the overall certainty of the evidence was reduced due to the weight of high risk of bias, low number of events, and high levels of heterogeneity.

## Conclusion

The present study indicates that FC administration in emergency settings may lead to a slight increase in in-hospital mortality. While there is low certainty of evidence to suggest that FC administration may reduce the need for RBC transfusions, it is probable that FC administration results in increased use of FFP transfusions, and is likely to result in a significant increase in PC transfusions with moderate certainty of the evidence. However, caution should be exercised in interpreting these findings due to the presence of imbalanced patient severity, high heterogeneity, and potential biases.

## Supplementary Information

**Additional file 1: Fig. S1.** Forest plots for secondary outcomes. **Fig. S2.** The forest plots of sensitivity analysis for each outcome. **Fig. S3.** The forest plots of subgroup analysis of trauma. **Fig. S4.** The forest plots of subgroup analysis of obstetrics. **Fig. S5.** Traffic light plots which reveal the evaluating the risk of bias for primary and secondary outcomes associated with individual RCTs based on the RoB2

## Data Availability

All relevant data are presented in the published manuscript.

## Abbreviations

CI: Confidence interval
FC: Fibrinogen concentrate
FFP: Fresh-frozen plasma
GRADE: Grading of Recommendations Assessment, Development and Evaluation
RCT: Randomized controlled trial

## References

[1] Rossaint R, Bouillon B, Cerny V, Coats TJ, Duranteau J, Fernandez-Mondejar E, Filipescu D, Hunt BJ, Komadina R, Nardi G (2016). The European guideline on management of major bleeding and coagulopathy following trauma: fourth edition. Crit Care.

[2] Ranucci M, Baryshnikova E, Castelvecchio S, Pelissero G (2013). Surgical, clinical outcome research G: major bleeding, transfusions, and anemia: the deadly triad of cardiac surgery. Ann Thorac Surg.

[3] Say L, Chou D, Gemmill A, Tunçalp Ö, Moller A-B, Daniels J, Gülmezoglu AM, Temmerman M, Alkema L (2014). Global causes of maternal death: a WHO systematic analysis. Lancet Glob Health.

[4] Hayakawa M (2017). Dynamics of fibrinogen in acute phases of trauma. J Intensive Care.

[5] Hiippala STMG, Vahtera EM (1995). Hemostatic factors and replacement of Major_Blood. Anesth Analg.

[6] Charbit BML, Samain E (2007). The decrease of fibrinogen is an early predictor of the severity of postpartum hemorrhage. J Thromb Haemost.

[7] de Lloyd L, Bovington R, Kaye A, Collis RE, Rayment R, Sanders J, Rees A, Collins PW (2011). Standard haemostatic tests following major obstetric haemorrhage. Int J Obstet Anesth.

[8] Fries D, Martini WZ (2010). Role of fibrinogen in trauma-induced coagulopathy. Br J Anaesth.

[9] Rahe-Meyer N, Levy JH, Mazer CD, Schramko A, Klein AA, Brat R, Okita Y, Ueda Y, Schmidt DS, Ranganath R (2016). Randomized evaluation of fibrinogen vs placebo in complex cardiovascular surgery (REPLACE): a double-blind phase III study of haemostatic therapy. Br J Anaesth.

[10] Winearls J, Wullschleger M, Wake E, Hurn C, Furyk J, Ryan G, Trout M, Walsham J, Holley A, Cohen J (2017). Fibrinogen Early In Severe Trauma studY (FEISTY): study protocol for a randomised controlled trial. Trials.

[11] Curry N, Foley C, Wong H, Mora A, Curnow E, Zarankaite A, Hodge R, Hopkins V, Deary A, Ray J (2018). Early fibrinogen concentrate therapy for major haemorrhage in trauma (E-FIT 1): results from a UK multi-centre, randomised, double blind, placebo-controlled pilot trial. Crit Care.

[12] Levy JH, Szlam F, Tanaka KA, Sniecienski RM (2012). Fibrinogen and hemostasis: a primary hemostatic target for the management of acquired bleeding. Anesth Analg.

[13] Li JY, Gong JS, Zhu F, Moodie J, Newitt A, Uruthiramoorthy L, Cheng D, Martin J (2018). Fibrinogen concentrate in cardiovascular surgery: a meta-analysis of randomized controlled trials. Anesth Analg.

[14] Fominskiy E, Nepomniashchikh VA, Lomivorotov VV, Monaco F, Vitiello C, Zangrillo A, Landoni G (2016). Efficacy and safety of fibrinogen concentrate in surgical patients: a meta-analysis of randomized controlled trials. J Cardiothorac Vasc Anesth.

[15] Wikkels A et al. Fibrinogen concentrate in bleeding paatients (Review). Cochrane Database Syst Rev. 2013. 10.1002/14651858.CD008864.pub2 PMC6517136 23986527

[16] Ng KT, Yap JL, Kwok PE (2020). The effect of fibrinogen concentrate on postoperative blood loss: a systematic review and meta-analysis of randomized controlled trials. J Clin Anesth.

[17] Collins PW, Cannings-John R, Bruynseels D (2017). Viscoelastometric-guided early fibrinogen concentrate replacement during postpartum haemorrhage: OBS2, a double-blined randomized controlled trial&nbsp;. Br J Anaesth.

[18] Innerhofer PFD, Mittemayr M (2017). Reversal of trauma-induced coagulopathy using first-line coagulation factor concentrates or fresh frozen plasma (RETIC): a single-cntre, parallel-group, open-label, randomised trial. Lancet Haematol.

[19] Stabler SN, Li SS, Karpov A, Vu EN. Use of fibrinogen concentrate for trauma-related bleeding: a systematic-review and meta-analysis. J Trauma Acute Care Surg. 2020. 10.1097/TA.0000000000002920 32890340

[20] Itagaki Y, Hayakawa M, Takahashi Y, Yamakawa K. Emergency administration of fibrinogen concentrate for hemorrhage a protocol for systematic review and meta-analysis. Medicine. 2021;100(10). 10.1097/MD.0000000000025099 PMC7969309 33725904

[21] Moher D, Liberati A, Tetzlaff J, Altman DG, Group P. Preferred reporting items for systematic reviews and meta-analyses: the PRISMA statement. PLoS Med. 2009;6(7):e1000097. 10.1371/journal.pmed.1000097 PMC2707599 19621072

[22] Ouzzani M, Hammady H, Fedorowicz Z, Elmagarmid A (2016). Rayyan-a web and mobile app for systematic reviews. Syst Rev.

[23] Sterne JAC, Savovic J, Page MJ, Elbers RG, Blencowe NS, Boutron I, Cates CJ, Cheng HY, Corbett MS, Eldridge SM (2019). RoB 2: a revised tool for assessing risk of bias in randomised trials. BMJ.

[24] Higgins JPT TJ CJ, Cumpston M, et al. Cochrane handbook for systematic reviews of interventions version 6.0 (updated July 2019). Cochrane. Available from www.training.org/handboo. 2019.

[25] Wan XWW, Liu J, Tong T (2014). Estimating the sample mean and standard deviation from the sample size, median, range and:or interquartile range. BMC Med Res Methodol.

[26] Luo D, Wan X, Liu J, Tong T (2018). Optimally estimating the sample mean from the sample size, median, mid-range, and/or mid-quartile range. Stat Methods Med Res.

[27] Copenhagen. The Nordic Cochrane Centre TCC. Review Manager (RevMan) [Computer program]. Version 5.4. 2019.

[28] Huedo-Medina TB, Sánchez-Meca J, Marín-Martínez F (2006). Assessing heterogeneity in meta-analysis-Q statistic or I2 index?. Psychol Methods.

[29] Santesso N, Glenton C, Dahm P, Garner P, Akl EA, Alper B, Brignardello-Petersen R, Carrasco-Labra A, De Beer H, Hultcrantz M (2020). GRADE guidelines 26: informative statements to communicate the findings of systematic reviews of interventions. J Clin Epidemiol.

[30] Akbari E, Safari S, Hatamabadi H (2018). The effect of fibrinogen concentrate and fresh frozen plasma on the outcome of patients with acute traumatic coagulopathy: a quasi-experimental study. Am J Emerg Med.

[31] Wilkkelso AJ, Edwards HM, Afshari A, et al. Pre-emptive treatment with fibrinogen concentrate for postpartum haemorrhage: randomized controlled trial. BJ Anaesth 2015;114(4):623–33. 10.1093/bja/aeu444 25586727

[32] Ziegler B, Bachler M, Haberfellner H, Niederwanger C, Innerhofer P, Hell T, Kaufmann M, Maegele M, Martinowitz U, Nebl C (2021). Efficacy of prehospital administration of fibrinogen concentrate in trauma patients bleeding or presumed to bleed (FIinTIC): a multicentre, double-blind, placebo-controlled, randomised pilot study. Eur J Anaesthesiol.

[33] Nascimento B, Callum J, Tien H, Peng H, Rizoli S, Karanicolas P, Alam A, Xiong W, Selby R, Garzon AM (2016). Fibrinogen in the initial resuscitation of severe trauma (FiiRST): a randomized feasibility trial. Br J Anaesth.

[34] Lucena LS, Rodrigues RDR, Carmona MJC, Noronha FJD, Oliveira HP, Lima NM, Pinheiro RB, Silva WAD, Cavalcanti AB (2021). Early administration of fibrinogen concentrate in patients with polytrauma with thromboelastometry suggestive of hypofibrinogenemia: a randomized feasibility trial. Clinics.

[35] Sabouri M, Vahidian M, Sourani A, Mahdavi SB, Tehrani DS, Shafiei E (2022). Efficacy and safety of fibrinogen administration in acute post-traumatic hypofibrinogenemia in isolated severe traumatic brain injury: a randomized clinical trial. J Clin Neurosci.

