# Supplementary material for: Emergency administration of fibrinogen concentrate for haemorrhage: systematic review and meta-analysis
Source: World J Emerg Surg. 2023 Mar 30;18:27. doi: 10.1186/s13017-023-00497-5 (PMC10061696; doi:10.1186/s13017-023-00497-5)
Supplement: Supplementary file 1 — Additional file 1: Fig. S1. Forest plots for secondary outcomes. Fig. S2. The forest plots of sensitivity analysis for each outcome. Fig. S3. The forest plots of subgroup analysis of trauma. Fig. S4. The forest plots of subgroup analysis of obstetrics. Fig. S5. Traffic light plots which reveal the evaluating the risk of bias for primary and secondary outcomes associated with individual RCTs based on the RoB2 [file 13017_2023_497_MOESM1_ESM.docx]

Supplementary Figure 1. Forest plots for secondary outcomes.

a. Blood loss within the first 24h


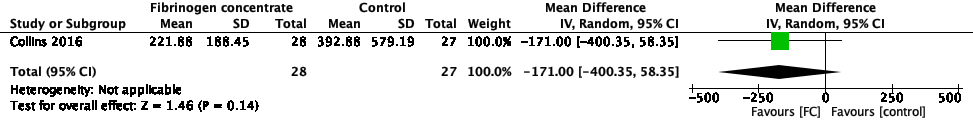


b. Thrombotic events


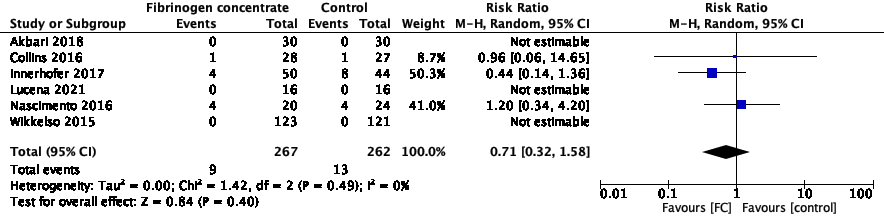


c. Multiple organ failure


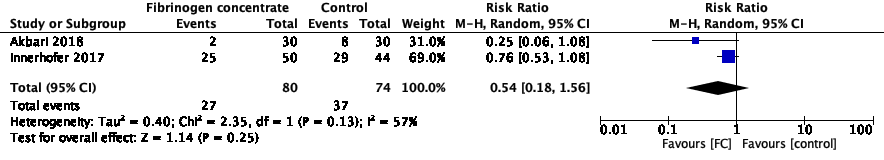


d. Length of ICU stay


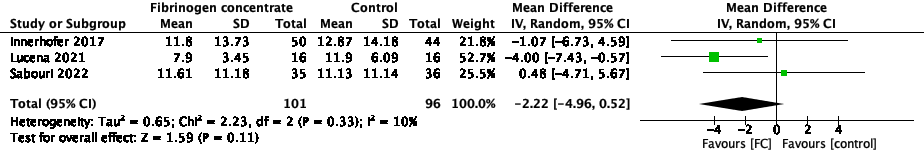


e. Length of hospital stay


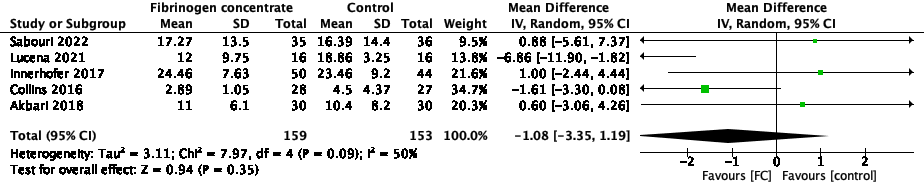


Supplement Figure 2. The forest plots of sensitivity analysis for each outcome.

a. In-hospital mortality


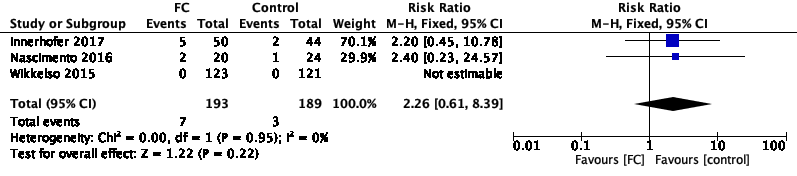


b. RBC transfusion in the first 24h


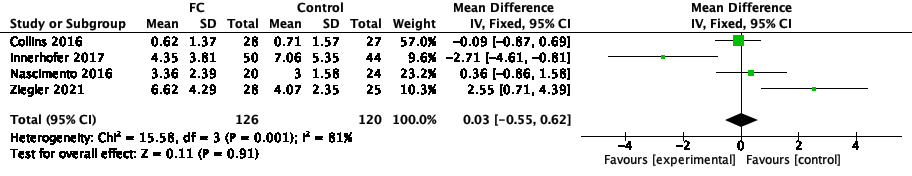


c. FFP transfusion in the first 24h


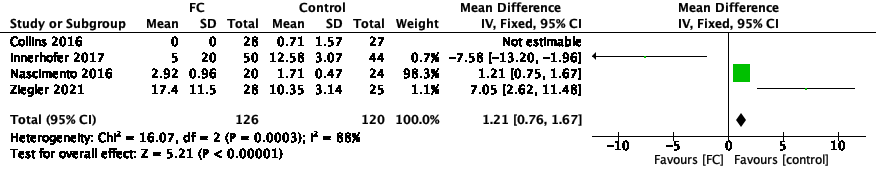


Supplement Figure 3. The forest plots of subgroup analysis of trauma.

1. In-hospital mortality


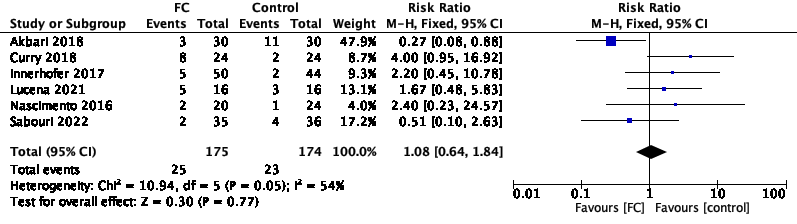


1. RBC transfusion in the first 24h


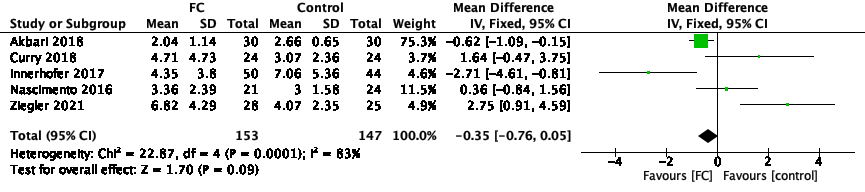


1. FFP transfusion in the first 24h


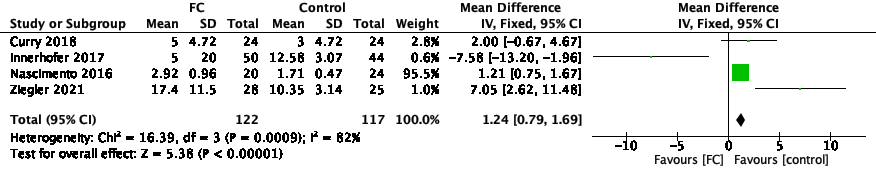


1. PC transfusion in the first 24h


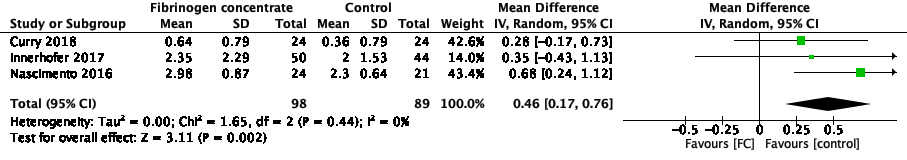


1. Thrombotic events


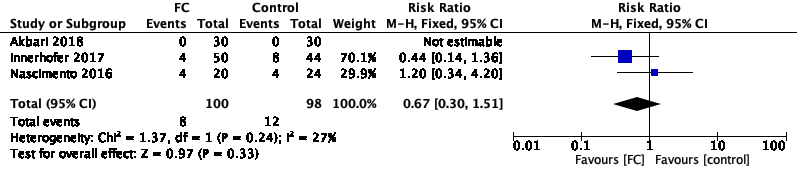


Supplement Figure 4. The forest plots of subgroup analysis of obstetrics.

1. RBC transfusion in the first 24h


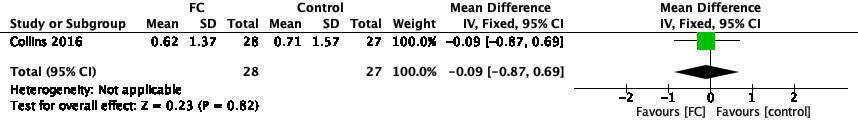


1. Thrombotic events


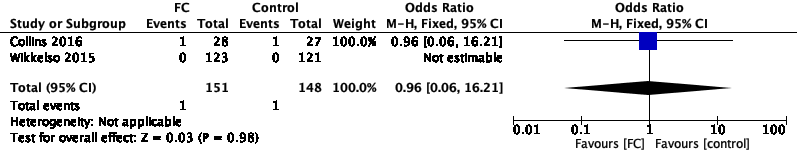


| **Supplementary Figure 5.** | **D1** | **D2** | **D3** | **D4** | **D5** | **Overall** |
| --- | --- | --- | --- | --- | --- | --- |
| In-hospital mortality |  |  |  |  |  |  |
| Lucena 2021 | High | Low | Low | Low | Low | High |
| Akbari 2018 | High | Low | Low | Low | High | High |
| Curry 2018 | Some concerns | Low | Low | Low | High | High |
| Innerhofer 2017 | Some concerns | Low | Low | Low | Low | Some concerns |
| Nascimeno 2016 | Low | Low | Low | Low | Low | Low |
| Wikkellso2015 | Low | Low | Low | Low | Low | Low |
| RBC transfusion in the first 24h |  |  |  |  |  |  |
| Ziegler 2021 | Low | Low | Some concerns | Low | Low | Some concerns |
| Akbari 2018 | High | Low | Low | Low | High | High |
| Curry 2018 | Some concerns | Low | Low | Low | High | High |
| Innerhofer 2017 | Some concerns | Low | Low | Low | Low | Some concerns |
| Nascimeno 2016 | Low | Low | Low | Low | Low | Low |
| Collins 2016 | Low | Low | Some concerns | Low | Low | Some concerns |
| FFP transfusion in the first 24h |  |  |  |  |  |  |
| Ziegler 2021 | Low | Low | Some concerns | Low | Low | Some concerns |
| Curry 2018 | Some concerns | Low | Low | Low | High | High |
| Innerhofer 2017 | Some concerns | Low | Low | Low | Low | Some concerns |
| Nascimeno 2016 | Low | Low | Low | Low | Low | Low |
| Collins 2016 | Low | Low | Some concerns | Low | Low | Some concerns |
| PC transfusion in the first 24h |  |  |  |  |  |  |
| Curry 2018 | Some concerns | Low | Low | Low | High | High |
| Innerhofer 2017 | Some concerns | Low | Low | Low | Low | Some concerns |
| Nascimeno 2016 | Low | Low | Low | Low | Low | Low |
| Blood loss within the first 24hrs |  |  |  |  |  |  |
| Collins 2016 | Low | Low | Some concerns | Low | Low | Some concerns |
| Thrombotic events |  |  |  |  |  |  |
| Lucena 2021 | High | Low | Low | Low | Low | High |
| Akbari 2018 | High | Low | Low | Low | High | High |
| Innerhofer 2017 | Some concerns | Low | Low | Low | Low | Some concerns |
| Nascimeno 2016 | Low | Low | Low | Low | Low | Low |
| Collins 2016 | Low | Low | Some concerns | Low | Low | Some concerns |
| Wikkellso2015 | Low | Low | Low | Low | Low | Low |
| Multiple organ failure |  |  |  |  |  |  |
| Akbari 2018 | High | Low | Low | Low | High | High |
| Innerhofer 2017 | Some concerns | Low | Low | Low | Low | Some concerns |
| Length of ICU stay |  |  |  |  |  |  |
| Sabouri 2022 | High | Low | Low | Low | Low | High |
| Lucena 2021 | High | Low | Low | Low | Low | High |
| Innerhofer 2017 | Some concerns | Low | Low | Low | Low | Some concerns |
| Length of hospital stay |  |  |  |  |  |  |
| Sabouri 2022 | High | Low | Low | Low | Low | High |
| Lucena 2021 | High | Low | Low | Low | Low | High |
| Akbari 2018 | High | Low | Low | Low | High | High |
| Innerhofer 2017 | Some concerns | Low | Low | Low | Low | Some concerns |
| Collins 2016 | Low | Low | Some concerns | Low | Low | Some concerns |
| D1: Bias arising from the randomization process. D2: Bias due to deviations from intended intervention. D3: Bias due to missing outcome data. D4: Bias in measurement of the outcome. D5: Bias in selection of the reported result. | | | | | | |
|  |  |  |  |  |  |  |
|  |  |  |  |  |  |  |
